# AGDIFF: Attention-Enhanced Diffusion for Molecular Geometry Prediction

**DOI:** 10.1021/acs.jcim.4c01896

**Published:** 2025-02-11

**Authors:** André Brasil Vieira Wyzykowski, Fatemeh Fathi Niazi, Alex Dickson

**Affiliations:** † Department of Biochemistry & Molecular Biology Michigan State University, East Lansing, Michigan 48824, United States; ‡ Department of Computational Mathematics, Science & Engineering Michigan State University, East Lansing, Michigan 48824, United States

## Abstract

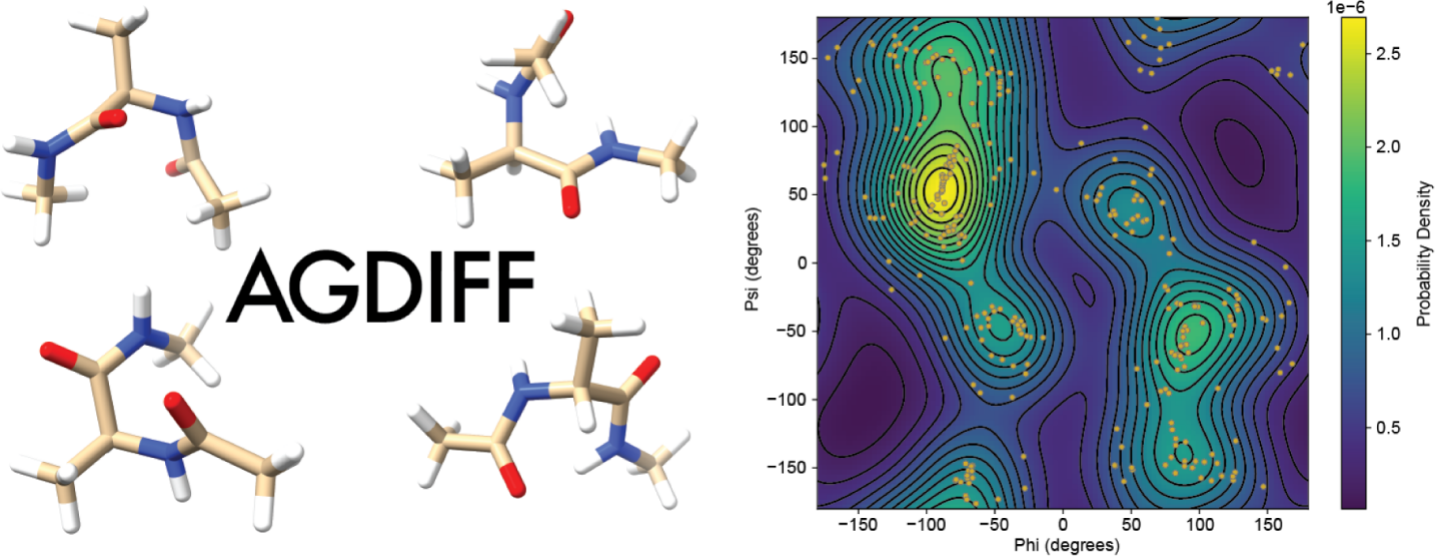

Accurate prediction of molecular geometries is crucial for drug discovery and materials science. Existing fast conformer prediction algorithms often rely on approximate empirical energy functions, resulting in low accuracy. More accurate methods like ab initio molecular dynamics and Markov chain Monte Carlo can be computationally expensive due to the need for evaluating quantum mechanical energy functions. To address this, we introduce AGDIFF, a novel machine learning framework that utilizes diffusion models for efficient and accurate molecular structure prediction. AGDIFF extends previous models (such as GeoDiff) by enhancing the global, local, and edge encoders with attention mechanisms, an improved SchNet architecture, batch normalization, and feature expansion techniques. AGDIFF outperforms GeoDiff on both the GEOM-QM9 and GEOM-Drugs data sets. For GEOM-QM9, with a threshold (δ) of 0.5 Å, AGDIFF achieves a mean COV-R of 93.08% and a mean MAT-R of 0.1965 Å. On the more complex GEOM-Drugs data set, using δ = 1.25 Å, AGDIFF attains a median COV-R of 100.00% and a mean MAT-R of 0.8237 Å. These findings demonstrate AGDIFF’s potential to advance molecular modeling techniques, enabling more efficient and accurate prediction of molecular geometries, thus contributing to computational chemistry, drug discovery, and materials design. https://github.com/ADicksonLab/AGDIFF

## 1 Introduction

The accurate prediction of molecular geometries is a fundamental challenge in computational chemistry. 3D structures of molecules, characterized by their atomic Cartesian coordinates, dictate their biological and physical properties, making them crucial for advancements in materials science and drug discovery. Traditional methods such as molecular dynamics (MD) and Markov chain Monte Carlo (MCMC) are theoretically robust. However, they are computationally demanding—particularly when combined with quantum mechanical energy functions, which are very expensive to evaluate.^1^ In addition, widely used cheminformatics toolkits like RDKit^2^ implement structure prediction algorithms that are efficient, but often struggle to generate accurate molecular geometries. A comparison of RDKit and a recently developed generative machine learning model called “GeoDiff”^3^ in conformer generation on the GEOM-Drugs data set^4^ reveals that RDKit has limited coverage of the reference conformational space and generates conformers of suboptimal quality. Further development is still needed for methods that can balance the needs for efficiency and accuracy.

The prediction of molecular geometries has evolved significantly over the decades, with each new approach addressing the limitations of its predecessors. Force field methods like MM2 and MM3 in the 1980s and 1990s were followed by semiempirical methods such as AM1 and PM3 in the 1990s and 2000s, which combined empirical data with quantum mechanical calculations for improved accuracy.^5−7^ Simultaneously, Monte Carlo simulations and simulated annealing were used for low-energy conformer searches.^8,9^ The 2000s saw the rise of quantum mechanical methods, including Hartree–Fock and DFT, which provided accurate 3D structures by solving the Schrödinger equation.^10,11^ In the 2010s, cheminformatics toolkits like RDKit and Open Babel emerged, facilitating 3D structure generation from SMILES strings.^2^ Early machine learning approaches, such as autoencoders,^12^ began exploring molecular structure prediction, laying the foundation for modern deep learning techniques in this field.

The general goal for structure prediction algorithms is to take the graph structure of a molecule as input (“2D” structure) and return a prediction of the 3D coordinates for each atom. The graph structure of a molecule is comprised of a set of nodes, one for each atom, and a set of edges, which show interactions between the atoms. The edges typically represent covalent bonds between atoms, but can also represent other things, such as spatial proximity. Each node can be annotated with a set of features, encoding information such as the element of the atom and the presence of an explicit charge. Similarly, edges are also given features, typically encoding the covalent bond type (single, double, aromatic, nonbonded, etc.). Here we denote a molecular graph with *n* nodes as *G*_*n*_, and the set of all molecular graphs as . The task of molecular structure prediction is then to develop a function (*f*) that maps .

These functions can be informed by earlier work in approaches developed for the prediction of molecular properties (). Early Quantitative Structure–Activity Relationship (QSAR) models relied on statistical methods and descriptors to correlate molecular structures with activities.^13^ Later, topological descriptors provided algebraic characterizations of molecular structures,^14^ and physicochemical descriptors improved predictive accuracy by considering properties like lipophilicity and solubility.^15^ The integration of machine learning algorithms, such as Support Vector Machines (SVM), Random Forest (RF), and Gradient Boosting Machines (GBM), further enhanced the predictive models by leveraging computational power to analyze complex data sets.^16^ These approaches have been particularly useful for predicting scalar molecular properties, including the internal energy, solubility constant, and partition coefficient (log P).^17,18^

All of the above approaches use projections of the graph onto an intermediate subspace of relevant features. However, the emergence of Graph Neural Networks (GNNs) revolutionized the field by providing a powerful framework for learning directly from molecular graph representations, eliminating the need for manual feature engineering and outperforming traditional methods in various predictive tasks.^19^ GNN-based approaches have shown promising results in various tasks within the domain of computational chemistry and drug discovery, as extensively reviewed elsewhere.^20^ One example is Attentive FP,^21^ a graph neural network architecture that leverages a graph attention mechanism for enhanced molecular representation in drug discovery. Attentive FP achieves state-of-the-art predictive performance for properties such as solubility, bioactivity, and lipophilicity. A prior study from our laboratory used the Geometric Scattering for Graphs method to automatically transform atomic features to a set of invariant graph-level features that could be used as inputs to a machine learning model.^22^ This obtained competitive results for prediction of log P,^23^ including in a blind, prospective study.^24^

GNN approaches have also been used for the  problem with the help of diffusion models. Diffusion models are a class of generative models that gradually transform simple random noise into complex data distributions. These models have achieved superior quality compared to state-of-the-art Generative Adversarial Networks (GANs).^25^ Latent Diffusion Models (LDMs), which operate in a learned latent space, can capture more complex patterns and generate high-quality samples. For example, Luo and Hu (2021)^26^ treated point clouds as particles in a thermodynamic system, utilizing a heat bath to facilitate diffusion from the original distribution to a noise distribution. These advancements showcase the versatility of diffusion models in handling complex data structures.

A key requirement for GNN-based diffusion models in molecular structure prediction is equivariance. That is, a transformation of the input should be equivalent to performing the same transformation on the output. Mathematically, for a given rotation matrix, R: . In a diffusion model, a GNN must transform input structures to a slightly “de-noised” version of the input. It is thus natural for these GNNs to be equivariant to displacements and rotations, in order to ensure adherence to underlying physical laws. A number of equivariant graph models have been developed that carry explicit geometric information with each node, and where all intermediate calculations satisfy the equivariance property.^27−30^ There has recently been a growing interest in the application of these models to molecular structure prediction in a diffusion model framework. Hoogeboom et al.^30^ introduced the E(3) Equivariant Diffusion Model (EDM) for 3D molecule generation, offering a model equivariant to Euclidean transformations that improves the quality and efficiency of generated molecular samples. Similarly, Liao and Smidt^27^ presented Equiformer, incorporating SE(3)/E(3)-equivariant features in a Transformer network for 3D atomistic graphs, demonstrating significant performance across data sets in the domain of 3D atomistic graphs.

An alternative strategy for achieving equivariance is to use the model to learn scalar “edge gradients” that are used to push and pull the atoms along interparticle displacement vectors. This simpler and effective approach is used by GeoDiff,^3^ a diffusion-based model for molecular conformation generation (summarized in Appendix B). Here, we build upon this approach to introduce AGDIFF, a diffusion model framework that incorporates a self-attention mechanism for efficient prediction of molecular structures. We extend GeoDiff by incorporating several enhancements to the global and local encoders, as well as the edge encoder, to improve the model’s expressiveness and adaptability. This direct  modeling approach circumvents the limitations inherent in indirect modeling and aims to capture the complex distribution of molecular geometries with high accuracy and efficiency.

In this work we first provide some background on diffusion models and their application to chemical systems. The AGDIFF model is described in detail, including a discussion of the model architecture, training and inference procedures. We present example inference trajectories and measure the overall quality of the structures generated for two commonly used data sets. We find that AGDIFF shows consistent improvement compared to previous GeoDiff results presented in Xu et al.^3^ We correlate sources of error with different molecular properties in the data sets and conclude with a discussion and outlook, including potential application of these algorithms in drug discovery.

## 2 Background on Diffusion Models

Diffusion models are a class of probabilistic generative models that learn to reverse a diffusion process to generate new data samples.^31^ Different formulations of these models include denoising diffusion probabilistic models (DDPMs),^32,33^ score-based generative models (SGMs),^34^ and models based on stochastic differential equations (score SDEs).^35^ As our work is specifically based on DDPMs, we will focus our discussion on this particular formulation.

**Figure 1:**
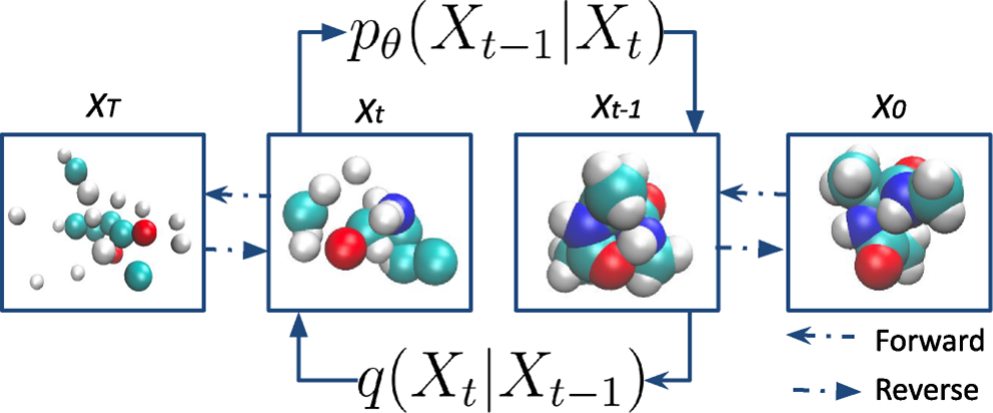
Illustration of the denoising diffusion probabilistic model (DDPM) approach for molecular conformation generation. The forward process involves adding noise to the molecular structure, transitioning from an initial structure *X*_0_ to a noisy version *X*_*T*_. The reverse process, modeled by _*p*θ_(*X*_*t–1*_|*X*_*t*_), helps reconstruct the original structure by gradually removing the noise.

DDPMs consist of two Markovian processes: a forward diffusion process and a reverse denoising process (Figure 1). In the forward diffusion process *q*, the noise is added to a data point *X* to generate a sequence of noisy points , ultimately resulting in a pure Gaussian distribution at time step *t*. In the reverse denoising process, a neural network is trained to iteratively remove the noise from the noisy data *X̃*_*T*_ back to the original clean distribution. The forward diffusion process is characterized by *q*(*X̃*_*t*_ | *X̃*_*t–1*_), which is the probability of the structure *X̃*_*T*_ and time *t*, given a structure *X̃*_*t–1*_ at time *t* – 1. This probability is given by a multivariate normal distribution:
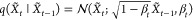
1where the notation  shows that the quantity *X* is distributed according to a Gaussian distribution with mean μ and variance *v*. The values of β_*t*_ follow a fixed variance schedule that controls how much noise is added, and should be selected to ensure that the final distribution follows a standard Gaussian.

This can be generally solved for the conditional distribution at any time step *t*:
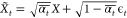
2where  and . In our work, the forward process has 5000 steps, with β_*t*_ defined as a sigmoidal function between β_min_ = 1*e –* 7 and β_max_ = 2*e –* 3. Specifically, 5000 values of *X* are linearly spaced between −6 and 6, these are used to define β_*t*_ as
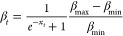
3

The reverse process aims to gradually denoise *X̃*_*T*_, arriving at an approximation (*X̑*_0_) of the original data using a neural network, whose parameters are denoted by θ. The probability distribution governing this process is referred to as *p*_θ_, which is generally formulated as
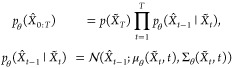
4where in our work, Σ_θ_(*X̃*_*t*_,*t*) is predefined and thus can be expressed as σ_*t*_^2^*I*, where . The mean μ_θ_(*X̃*_*t*_,*t*) is computed using the predicted noise ϵ_θ_(*X̃*_*t*_,*t*) from the neural network as follows:
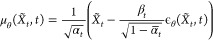
5

The goal during training is to minimize the negative log-likelihood of the source data as our objective function. However, since calculating–log(*p*_*θ*_(*X*)) is intractable, DDPMs instead train the neural network by maximizing the Evidence Lower Bound (ELBO), which approximates the log-likelihood.^33^ After simplifying, this training objective can ultimately be described as
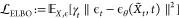
6where  refers to a weight term. By using *X̃*_*T*_ from eq 2, the training objective function can also be rewritten as
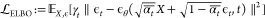
7

To infer samples from our trained model, we begin by drawing a sample from Gaussian prior, . A slightly denoised state *X̑*_*T-1*_ is then calculated as
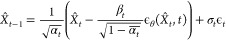
8

This process is repeated for *t* iterations, progressively sampling *X̑*_*T–1*_ at each step, until the final data state *X̑*_0_ is obtained.

## 3 Methods

**Figure 2:**
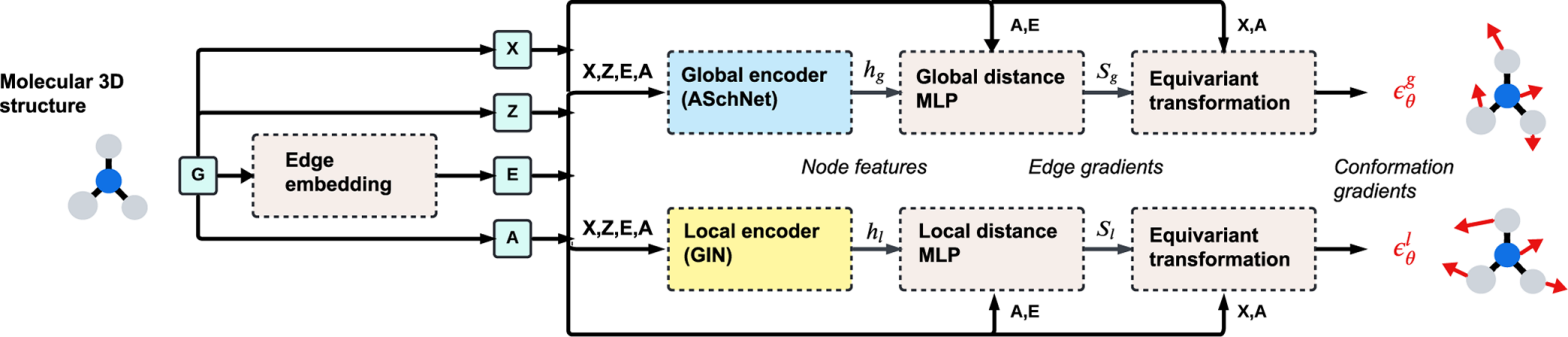
Schematic overview of the AGDIFF workflow. The input to the model is the 3D structure of the molecule, which includes information about atom types *Z*, bond indices *A*, bond types *E*, and atom coordinates *X*. This information is passed to two separate encoder streams, “global” and “local”, with the latter receiving a reduced set of edge indices focusing on local covalent bonds. Each stream uses a multilayer perceptron (MLP) and an equivariant transformation to predict conformation gradients, or directions attached to each atom that are used for coordinate updates during the reverse process.

The GeoDiff^3^ method employed a dual-encoder architecture for node embedding comprised of a SchNet-based^36^ global encoder that captures the overall geometric arrangement and long-range atomic interactions, a local encoder (GIN) that processes fine-grained details and local chemical environments. Edge features are generated with an MLP-based edge encoder that processes edge attributes such as bond types and lengths. AGDIFF extends and improves upon these foundational elements by introducing learnable activation functions, attention mechanisms, adaptive scaling modules, dual pathway processing, enhanced CFConv layers, batch normalization, and feature expansion (Figure 2).

The global encoder, operating on the complete molecular graph, processes node positions (*X*), attributes (*Z*), edge indices (*A*), and edge attributes (*E*) to produce a set of features for each node (*h*_*g*_) that encode the molecule’s overall structure. The local encoder, focuses on the local environment of each atom and operates on a smaller set of edges. The “local” set of edges is determined starting with the set of covalent bonds, with edge types discriminating between single, double, triple and aromatic bonds. This is supplemented with additional edges for atoms that are separated by two or three hops in the molecular graph, each given a distinct edge type. The “non-local” edges are determined using spatial proximity, where all pairs of atoms within a cutoff (10 Å) are joined with an edge, again given a distinct edge type. Both local and nonlocal edges are passed to the global encoder and only local edges are passed to the local encoder.

Both encoders are run through separate MLP modules that transform the node features into “scores” for each edge (*S*_*g*_ for the global branch and *S*_*l*_ for the local branch). These are separately transformed into vectors for each atom that, when successfully trained, will point in the direction that best lowers the loss function defined in eq 6. Following previous work,^3^ separate loss values are computed for the global and local perturbations, which are combined as . During inference the global and local gradients are mixed as . In the following subsections, we will delve into the details of each encoder and its respective architectures, highlighting the key components and enhancements introduced in AGDIFF.

### 3.1 Global Encoder

**Figure 3:**
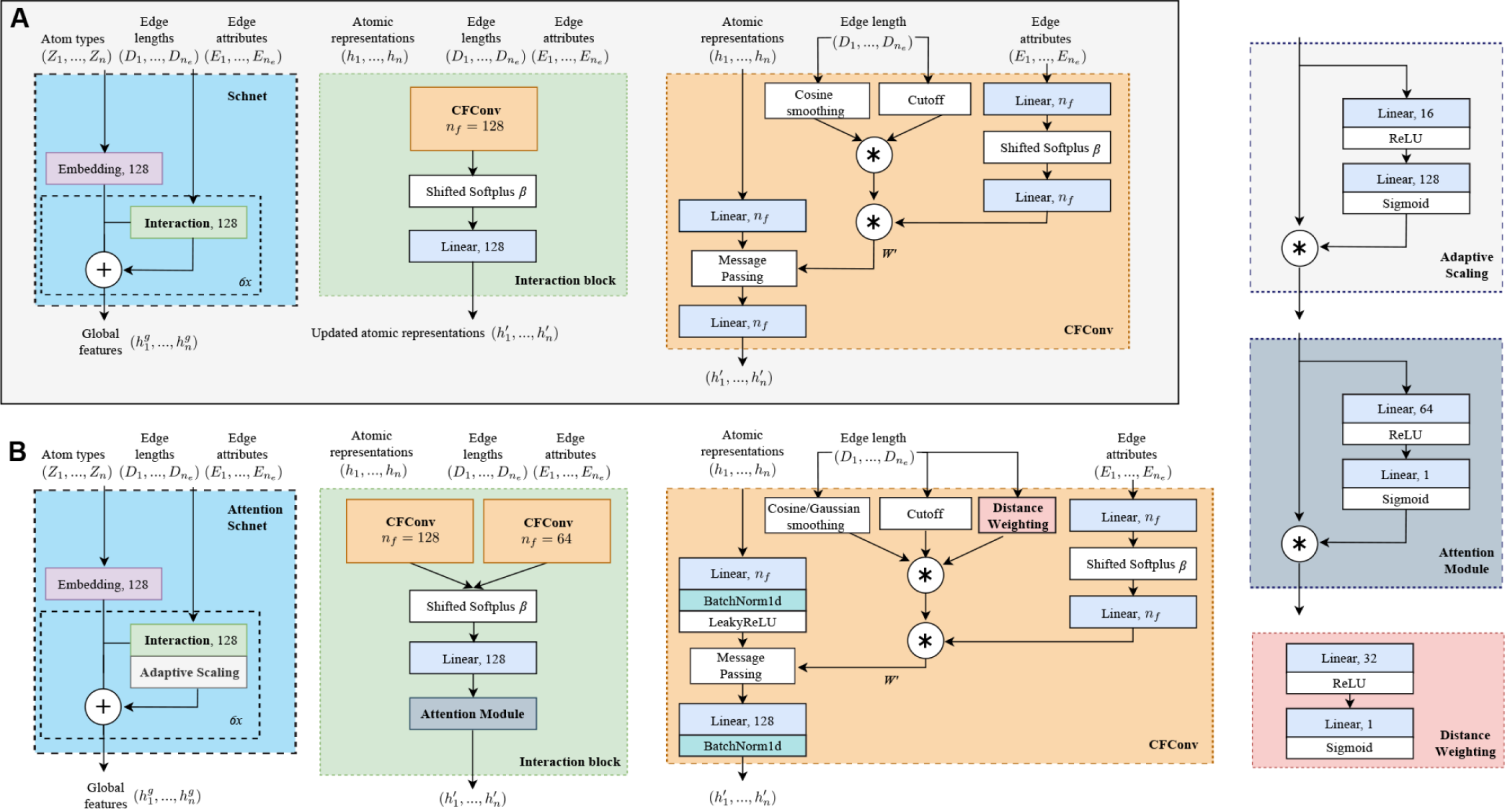
Enhancement of the global SchNet encoder. A) The SchNet implementation used in previous work. B) The Attention SchNet architecture. Comparison of the two panels shows how the modifications—including learnable activation functions, attention mechanisms, adaptive scaling modules, dual pathway processing, and enhanced CFConv layers—are integrated into the SchNet framework. In both panels, * denotes element-wise multiplication, **+** denotes element-wise addition, and numbers like 128 represent the dimensionality of the output feature vectors (per node) after each layer.

In this work, we propose an enhanced version of the SchNet architecture, a deep learning framework introduced by Schütt et al.^36^ for learning molecular representations. SchNet is a graph neural network that utilizes continuous-filter convolutions (CFConv) and interaction blocks to capture the complex interactions between atoms in a molecule. In comparison with other graph neural network models such as Nequip,^37^ the SchNet model has roughly 9-fold fewer parameters and is over 5 times faster in a molecular simulation context.^38^Figure 3A shows the SchNet architecture previously used to generate molecular conformations in ref^3^. The architecture consists of an embedding layer (creating 128 features per node) followed by multiple interaction blocks, each comprised of a CFConv layer, a softplus and an atom-wise dense layer. The outputs of the interaction blocks are sequentially added to the node features using a residual connection. The CFConv layer applies a filter-generating network to the atom embeddings and aggregates the results based on the interatomic distances. By stacking multiple interaction blocks, SchNet enables multiple rounds of message passing and feature extraction, allowing the model to learn intricate patterns and relationships within the molecular structure. Here we aim to improve the expressiveness of invariant GNN models, such as SchNet, while maintaining the advantages of their relatively lightweight neural network structure.

We introduce several modifications to the SchNet architecture that are shown in Figure 3B. These enhancements are designed to be computationally efficient, ensuring that the model remains fast and scalable, which is particularly important when considering the time-consuming nature of diffusion model steps. The proposed modifications include the incorporation of attention mechanisms, and adaptive scaling modules, enabling the model to dynamically focus on relevant features and interactions. Additionally, we introduce dual pathway processing and enhanced CFConv layers to increase the model’s expressiveness and learning capacity. Each enhancement is discussed in detail below:

(1) Enhanced CFConv Layers: We modified the CFConv layer to improve its learning capacity, robustness and training stability. Batch normalization is applied to the feature maps to stabilize training and speed up convergence. A LeakyReLU activation is used before the message passing layer, which maintains a small gradient for inactive units, preventing them from becoming completely inactive during training. Additionally, we integrate a learnable distance weighting (pink box in Figure 3B) into the edge weight calculation stream to allow for more complex distance dependencies between atoms.
  To speed up convergence during training, the distance weighting module operates in conjunction with a Gaussian-based distance function and a cutoff:
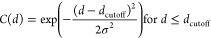
9where *d* represents the interatomic distance, *d*_cutoff_ is the predefined maximum distance for interactions, and σ is set to *d*_cutoff_ to control the spread of the Gaussian envelope. Here, *d*_cutoff_ is set to 10.0 Å. All edge weights are set to zero for *d* > *d*_cutoff_ to ensure that physically irrelevant interactions are not considered. These cutoff-based weights are then combined with the learnable weights to produce the final weights used in the convolution, enhancing the model’s ability to prioritize relevant interactions dynamically.
(2) Enhanced interaction block: To increase the expressiveness of the interaction blocks, we introduce a dual pathway processing scheme. Each interaction block now consists of two parallel CFConv layers with separate filter-generating networks. The motivation behind this design is to allow the model to learn different aspects of the molecular data simultaneously. One pathway uses a higher number of hidden dimensions for the graph convolution operation, and one uses a lower number. The rationale for incorporating both pathways is grounded in their complementary strengths, with the standard CFConv (128 filters) enabling the identification of long-range dependencies and high-order interactions, and the reduced CFConv (64 filters) capturing more general patterns and interactions that might be missed by the more complex pathway.
  The outputs of the CFConv layers are concatenated and then passed through a *ShiftedSoftplus* activation layer. This introduces a set of learnable parameters that enable the model to dynamically adapt the activation function’s response curve for each element during training. These outputs are then concatenated and processed using an attention mechanism to effectively integrate the information from both pathways by focusing on the most relevant features. This approach is a simplified idea of a multihead attention mechanism,^39^ where multiple heads operate in parallel, each learning to focus on different parts of the input data. Here, the attention module learns a set of weights using a neural network comprising two linear layers (blue box in Figure 3B). The first linear layer reduces the dimensionality by half and applies a ReLU activation function. The second linear layer further processes these features, followed by a Sigmoid activation function to produce the final attention weights. This allows the model to scale feature values in an atom-wise manner, affecting the subsequent message passing steps.
(3) Adaptive Feature Scaling: We integrate adaptive scaling modules into the main encoder loop of the SchNet architecture that dynamically scale the features based on global information. This module computes scaling factors for each feature channel, which are then combined with the original features using element-wise multiplication. This adaptive scaling mechanism enables the model to emphasize or suppress certain features based on their relevance to the global molecular representation. By dynamically adjusting the feature scales, the model can adapt to different molecular properties and prioritize the most informative features for the given task.

### 3.2 Local Encoder

**Figure 4:**
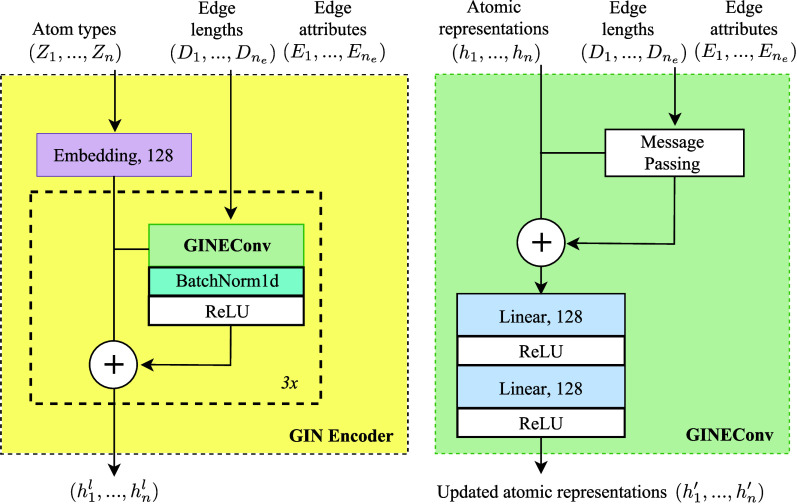
Architecture of the local encoder used in this work. This is similar to that used in ref^3^, except that a batch normalization is used to stabilize the training.

The local encoder, based on the GINEncoder from GeoDiff,^3^ utilizes layers from GINEConv^40^ to process node features, edge indices, and edge attributes. We introduce batch normalization layers after each convolution in the GINEncoder to stabilize training and improve convergence. As illustrated in Figure 4, the GINEncoder begins with node type embeddings and passes them through a series of GINEConv layers. Each GINEConv layer performs a message passing step that aggregates and updates node features, these are recombined with the additional node features with a residual connection. The node features are then passed through a two-layer MLP with ReLU activation. Batch normalization and ReLU activation are applied after each GINEConv layer, except for the last layer. The final output represents the updated node features.

### 3.3 Edge Encoder

**Figure 5:**
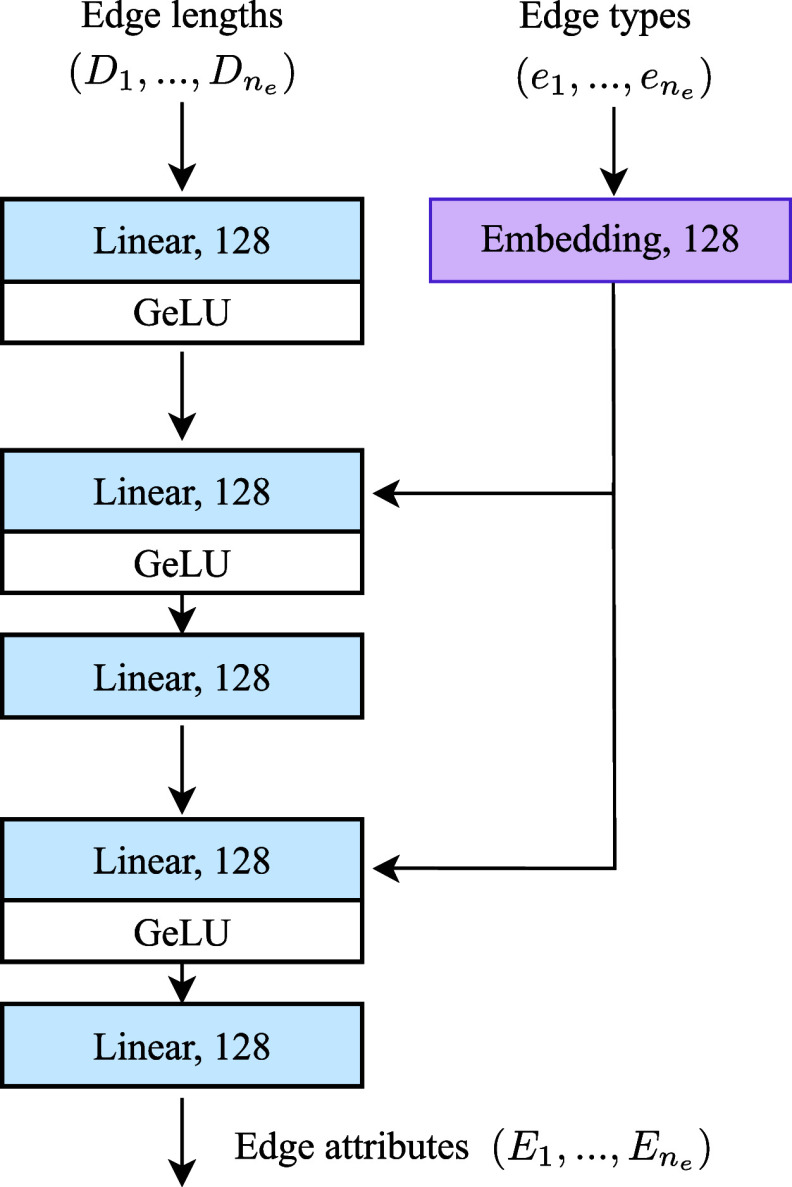
Architecture of the MLP Edge Encoder. This encoder processes edge types and lengths, producing feature vectors that capture both geometric and chemical information on the molecular graph. Each edge is encoded independently, and the numbers specified in the Linear and Embedding layers show the number of features used to represent each edge.

The edge encoder is responsible for capturing the geometric and chemical information on the molecular graph by processing edge attributes such as edge types and edge lengths. The edge types include different covalent bond types (e.g., single, double, triple, aromatic) as well as graph proximity (2-hop and 3-hop) and spatial proximity. The edge encoder takes both edge lengths and edge types as input and outputs a feature vector representing the encoded edge information (Figure 5). Our work expands upon the edge encoder proposed by GeoDiff,^3^ which employed a multilayer perceptron (MLP) to process edge lengths and combined them with bond embeddings using element-wise multiplication.

Our enhanced MLP Edge Encoder first uses a linear layer to expand the edge lengths to a higher-dimensional space. These are concatenated with edge type embeddings which are also learned using an embedding layer. The concatenated features are passed through a multilayer perceptron to introduce nonlinearity to allow the model to learn more abstract representations. The Gaussian error Linear Unit (GeLU) activation function is used in MLP_1_ for smooth and efficient activation.^41^ The processed edge features are then reintegrated with the bond type embeddings and passed through a second MLP. In the forward pass of the model, the MLP Edge Encoder is applied to all edges in the molecular graph, regardless of their context. The encoded edge attributes are then utilized by the subsequent components of the model, such as the local and global encoders discussed above.

### 3.4 Data Sets

The data sets used in this study include GEOM-QM9 and GEOM-Drugs.^4^ These provide a diverse range of molecular structures to comprehensively assess the performance and generalization capabilities of our model, and a concrete benchmark for comparison to other approaches.

The GEOM-QM9 data set is a subset of the larger QM9 data set, consisting of small organic molecules with up to 9 heavy atoms (C, O, N, and F). The data set includes optimized 3D geometries computed using density functional theory (DFT), making it valuable for training models aimed at predicting molecular conformations. The data set is split into training, validation, and test sets, with 40000, 5000, and 200 molecules, respectively.

The GEOM-Drugs data set contains larger and more complex drug-like molecules, providing a greater challenge due to increased molecular size and diversity. The data set is split into training, validation, and test sets, with 39000, 5000, and 200 molecules, respectively.

### 3.5 Training Procedure

The Adam optimizer is used with a learning rate of 1 × 10^–3^, no weight decay, and β_1_ and β_2_ values set to 0.95 and 0.999, respectively. A learning rate scheduler of type’plateau’ is applied, reducing the learning rate by a factor of 0.6 if the validation performance does not improve for 10 consecutive validations. Training is conducted using batches of 64 for GEOM-QM9 and 32 for GEOM-Drugs. To ensure stable training, gradients are clipped to a maximum norm of 10,000 for GEOM-QM9 and 30,000 for GEOM-Drugs. Model performance is monitored on the validation set every 5,000 iterations to ensure effective learning and prevent overfitting. A complete set of the model parameters and configurations is given in Appendix A.

## 4 Results

### 4.1 Analysis of Alanine-Dipeptide Diffusion Trajectories

**Figure 6:**
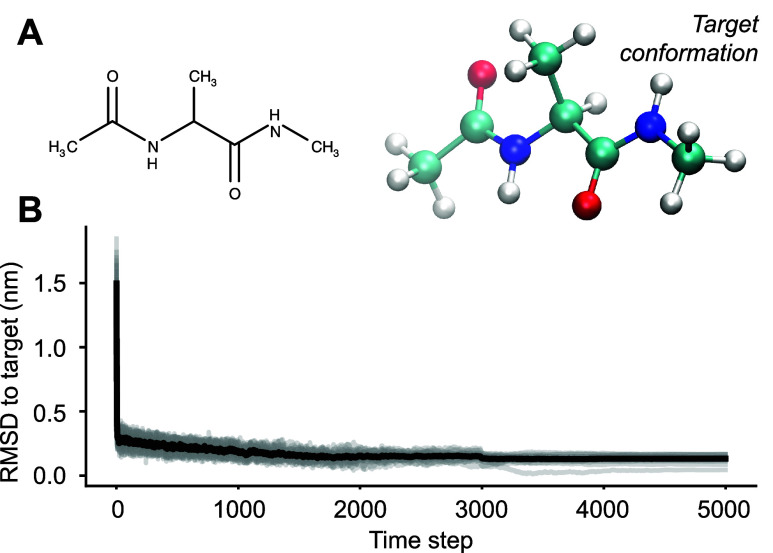
A) The 2D (left) and 3D (right) structure of alanine dipeptide. Note that this is one of many conformers. B) RMSD to the 3D conformer from A measured for 20 different assembly simulations (gray). The average is shown as a thick black line.

To understand the AGDIFF model’s refinement process over the course of an inference trajectory, we first study the structure generation trajectories of alanine dipeptide. Alanine dipeptide is a common benchmarking system in computational chemistry with a well-known conformational energy landscape (Figure 6A). Figure 6B shows the RMSD as a function of the 5000 steps for a set of 20 trajectories, as well as the mean RMSD. The high initial RMSD values (approximately 1.5 nm) decrease quickly in the first few time steps, followed by a slower more gradual improvement. By approximately 3000 steps, the structures are largely locked in, with only minimal changes to follow.

**Figure 7:**
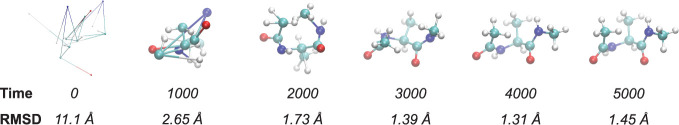
An example alanine dipeptide diffusion trajectory. RMSD is calculated to the single reference structure in Figure 6A using heavy atoms. The bonds shown in each frame are consistent with the final covalent bonding pattern and help track the progress of assembly.

Figure 7 visualizes the molecular conformations at different timesteps for a representative trajectory. The *t* = 0 structure shows randomly distributed atoms within a broad envelope. By *t* = 1000 the atoms have consolidated, but have not yet found their binding partners. The *t* = 2000 structure shows atoms in the correct general area, with reasonable bond lengths between atoms, but with incorrect local geometries in the angles and torsions. For *t* ≥ 3000, the atoms have settled into a reasonable conformation, with steadily improving geometric properties over time. As many structures are possible, the RMSD is not an absolute measure of structural quality, but is only meant to act as a rough indicator of assembly progress.

**Figure 8:**
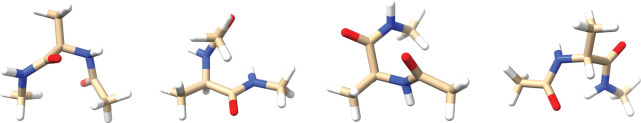
Generated conformations for alanine dipeptide using the AGDIFF model.

**Figure 9:**
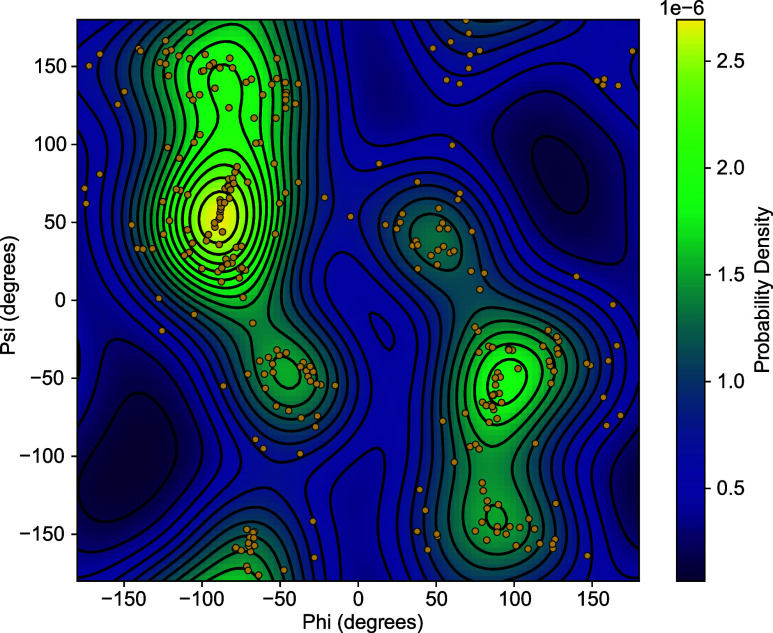
Ramachandran plot for alanine dipeptide, showing the probability density as a function of dihedral angles Φ and Ψ. The plot is overlaid with 200 generated conformers (orange dots) from the AGDIFF model. The color gradient represents the probability density, with yellow/green indicating high probability (stable conformations) and dark blue/black indicating low probability (less likely conformations). The free energy landscape (background) was obtained by calculating the density of the dihedral angles Φ and Ψ (orange points) using a Gaussian kernel density estimator (KDE) including periodic images of the points. This closely resembles other Ramachandran plots of alanine dipeptide in vacuum (e.g., ref^42^ and ref.^43^).

By running multiple independent assembly trajectories, we can generate many possible alanine dipeptide conformations. Figure 8 shows four different assembled conformations of alanine dipeptide created with different initial conditions and random seeds. To more thoroughly assess the quality and diversity of the conformations generated by AGDIFF, we analyze the Ramachandran plot of alanine dipeptide, which depicts the probability density as a function of the dihedral angles Φ and Ψ (Figure 9).

The high probability regions of the plot are largely consistent with prior studies of alanine dipeptide using quantum mechanical geometry optimizations (e.g., Figure 2 of ref^43^). The highest probability region on the map is the C7_eq_ region, around (ϕψ) = (−60,60). This coverage extends well to the C5 and β regions, at (ϕψ) = (−150,150) and (−60,150), respectively. The highest probability region on the  0 side was (100,–50), also consistent with previous work. Overall, the coverage of the high-free-energy areas demonstrates the accuracy of AGDIFF in identifying a variety of stable molecular conformations.

Moreover, the conformers are well-distributed across the entire free energy landscape, including regions of lower probability. For instance, the small cluster of points around (ϕψ) = (60,150) were also previously shown to be a local free energy minimum, although globally unstable. This highlights AGDIFF’s ability to explore the entire conformational space of a molecule, which is valuable for chemists studying the conformational ensembles. However, it also underscores the need to draw ensembles of structures from the model in order to distinguish which structures are the most common. It also highlights the importance of using multiple target structures when assessing the accuracy of the model predictions.

### 4.2 Evaluation of Generated Conformation Quality and Diversity

We now shift toward a more broad-scale assessment of the model on the testing portions of the QM9 and DRUGS data set discussed above. We used two primary metrics, Coverage (COV) and Matching (MAT), as introduced by^44^, to compare a set of generated conformations (*S*_*g*_) with a reference set (*S*_*r*_). These metrics are defined as follows:
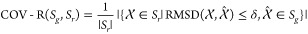
10
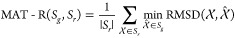
11where δ is a predefined RMSD threshold. COV-R measures the proportion of reference conformations covered by the generated set, indicating diversity. A higher COV-R score suggests a better coverage of the reference conformational space. MAT-R calculates the average RMSD between each reference conformation and its nearest neighbor in the generated set, indicating quality. A lower MAT-R score implies higher quality generated conformations. We also use precision counterparts, COV-P and MAT-P, which swap the roles of generated and reference sets. COV-P measures the proportion of generated conformations close to any reference conformation, while MAT-P computes the average RMSD between each generated conformation and its nearest reference conformation, focusing on realism and plausibility.

The RMSD threshold δ is data set-specific, based on previous studies.^44,45^ For the QM9 data set, δ is set to 0.5 Å, and for the Drugs data set, δ is 1.25 Å. These thresholds balance precise matching with tolerance for conformational variations. In our experiments, we generate a set of conformations *S*_*g*_ that is twice the size of the reference set *S*_*r*_ for each molecule. This allows us to generate structures next to each reference, considering that not all reference structures will be of the same probability. It also follows previous work, allowing a precise comparison with results from GEODIFF.^3^ GEODIFF was trained with two types of modified ELBO, named “alignment” and “chain-rule” approaches. We denote models learned by these two objectives as GEODIFF-A and GEODIFF-C respectively, consistent with their nomenclature in ref^3^.

Table 1 presents the results on the GEOM-QM9 data set.^4^ Our AGDIFF model outperforms the GEODIFF-A and GEODIFF-C baselines across all metrics^1^. To isolate the contributions of individual components we introduced two variants: AGDIFF-EDGE (only edge encoder enhancements) and AGDIFF-GLOBAL (only global encoder enhancements).^2^ This approach quantifies the impact of each component on AGDIFF’s overall performance.

**Table 1:** Results on the **GEOM-QM9** Dataset (T = 5000 Steps)

	COV-R (%) ↑		MAT-R (Å) ↓	
Models	Mean	Median	Mean	Median
GraphDG	73.33	84.21	0.4245	0.3973
CGCF	78.05	82.48	0.4219	0.3900
ConfVAE	77.84	88.20	0.4154	0.3739
GeoMol	71.26	72.00	0.3731	0.3731
ConfGF	88.49	94.31	0.2673	0.2685
GEODIFF-A	90.54	94.61	0.2104	0.2021
GEODIFF-C	90.07	93.39	0.2090	0.1988
GADIFF	90.50	93.33	0.2142	0.2140
AGDIFF-EDGE	91.79	94.84	0.2071	0.2045
AGDIFF-GLOBAL	92.30	95.75	0.2200	0.2060
AGDIFF	**93.08**	**96.25**	**0.1965**	**0.1919**

	COV-P (%) ↑		MAT-P (Å) ↓	
	Mean	Median	Mean	Median
GraphDG	43.90	35.33	0.5809	0.5823
CGCF	36.49	33.57	0.6615	0.6427
ConfVAE	38.02	34.67	0.6215	0.6091
ConfGF	46.43	43.41	0.5224	0.5124
GEODIFF-A	52.35	50.10	0.4539	0.4399
GEODIFF-C	52.79	50.29	0.4448	0.4267
GADIFF	**59.52**	**56.41**	**0.4070**	**0.3579**
AGDIFF-EDGE	56.49	55.22	0.4211	0.3897
AGDIFF-GLOBAL	55.12	52.63	0.4377	0.4224
AGDIFF	56.62	54.69	0.4156	0.3987

For the coverage metric (COV-R), AGDIFF achieves the highest mean (93.08%) and median (96.25%), surpassing all other models. AGDIFF-GLOBAL (mean: 92.30%, median: 95.75%) slightly outperforms AGDIFF-EDGE (mean: 91.79%, median: 94.84%), and both models show a slight improvement over the GEODIFF results. In terms of matching (MAT-R), again AGDIFF achieves the lowest values (mean: 0.1965 Å, median: 0.1919 Å), demonstrating superior performance in reducing RMSD. In this measure, AGDIFF-EDGE (mean: 0.2071 Å, median: 0.2045 Å) performs slightly better than AGDIFF-GLOBAL (mean: 0.2200 Å, median: 0.2060 Å), although both models are in the neighborhood of previous GEODIFF results. The precision metrics (COV-P and MAT-P) further confirm AGDIFF’s enhancements over GEODIFF. AGDIFF achieves a higher mean COV-P (56.62%) and lower mean MAT-P (0.4156 Å). Notably, AGDIFF-EDGE shows strong performance in precision, with the highest median COV-P (55.22%) and lowest median MAT-P (0.3897 Å) among all variants, highlighting the edge encoder’s importance for precise predictions. These results demonstrate that while both encoder enhancements contribute to AGDIFF’s performance, they excel in different aspects, and across almost all measures, work better when combined. We note that GADIFF,^50^ another attention-enhanced SchNet encoder-based method published recently, outperforms AGDIFF in precision metrics for QM9.

Table 2 shows the results on the GEOM-Drugs data set, which contains larger and more complex molecules compared to QM9. AGDIFF again consistently outperforms the GEODIFF-A and GEODIFF-C baselines across all metrics. Notably, AGDIFF achieves a median COV-R of 100.00%, indicating that it generates conformations that fully cover the reference conformational space for the majority of molecules in the data set. The lower MAT-R values (mean: 0.8237 Å, median: 0.8058 Å) demonstrate the high quality of the generated conformations. The precision metrics, COV-P and MAT-P, also showcase the superior performance of AGDIFF, with higher COV-P values and lower MAT-P values compared to the GEODIFF baselines. We again note that GADIFF^50^ shows improved performance in comparison to AGDIFF in all metrics for the GEOM-Drugs data set besides the median COV-R, where both methods achieved 100.0%.

**Table 2:** Results on the **GEOM-Drugs** Dataset (T = 5000 Steps)

	COV-R (%) ↑		MAT-R (Å) ↓	
Models	Mean	Median	Mean	Median
GraphDG	8.27	0.00	1.9722	1.9845
CGCF	53.96	57.06	1.2487	1.2247
ConfVAE	55.20	59.43	1.2380	1.1417
GeoMol	67.16	71.71	1.0875	1.0586
ConfGF	62.15	70.93	1.1629	1.1596
GEODIFF-A	88.36	96.09	0.8704	0.8628
GEODIFF-C	89.13	97.88	0.8629	0.8529
GADIFF	**95.37**	**100.00**	**0.6209**	**0.5960**
AGDIFF	91.31	**100.00**	0.8237	0.8058

	COV-P (%) ↑		MAT-P (Å) ↓	
	Mean	Median	Mean	Median
GraphDG	2.08	0.00	2.4340	2.4100
CGCF	21.68	13.72	1.8571	1.8066
ConfVAE	22.96	14.05	1.8287	1.8159
ConfGF	23.42	15.52	1.7219	1.6863
GEODIFF-A	60.14	61.25	1.1864	1.1391
GEODIFF-C	61.47	64.55	1.1712	1.1232
GADIFF	**74.59**	**80.73**	**0.9282**	**0.8696**
AGDIFF	64.34	69.36	1.1316	1.0805

**Figure 10:**
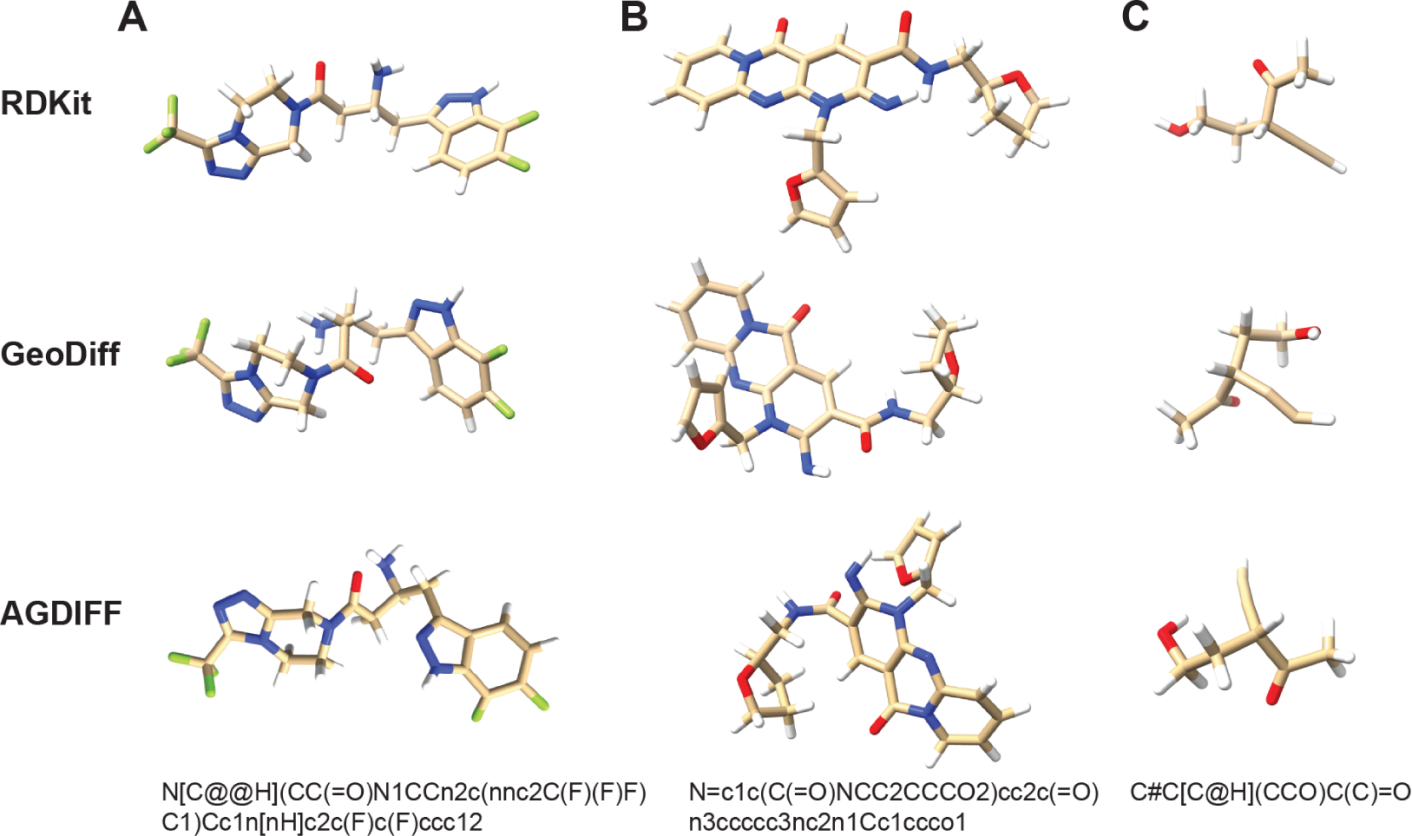
Generated conformations for drug-like molecules from the GEOM-Drugs data set. Each column displays 3D structures for a given molecule, determined using RDKit (top), GeoDiff (middle) and AGDIFF (bottom), with its corresponding SMILES string representation shown below. Molecules are shown in a licorice representation where carbon is tan, oxygen is red, nitrogen is blue, hydrogen is white, and fluorine is green.

The Drugs data set contains larger and more challenging molecules than QM9. Figure 10 shows predicted structures for three representative molecules in the data set. These examples demonstrate the diversity and complexity of the molecules, including conjoined aromatic ring structures, polyfluorinated compounds, and a diversity of bond types, including C–C triple bonds. The predicted structures show some revealing differences. For RDKit, the local geometry is mostly flawless, but some longer range interactions (such as the two adjacent polar −NH hydrogens in panel B) are clearly suboptimal. In the diffusion approaches there are some noticeable imperfections: some aromatic ring structures in a) and b) are not perfectly planar, and the methylidyne group in c) is nonlinear. But overall this showcases the ability of the AGDIFF model to generate realistic and chemically valid conformations for more complex, drug-like compounds.

### 4.3 Prediction Accuracy as a Function of Molecular Flexibility

**Figure 11:**
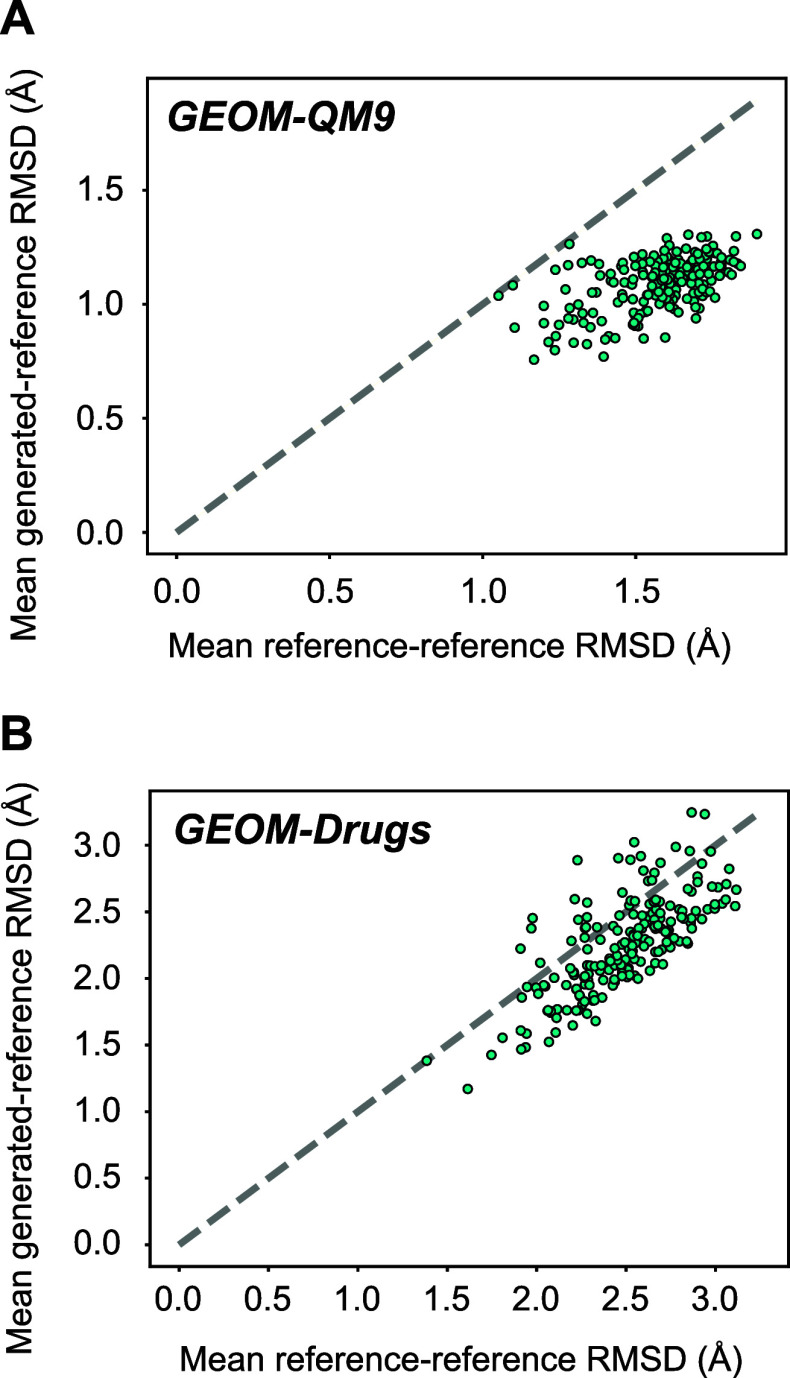
Scatter plot comparing the mean RMSD of each generated structure to all of the reference structures (vertical axis) with the mean of all reference-reference RMSDs (horizontal axis). Each point represents a single compound in the GEOM-QM9 data set (A) or the GEOM-Drugs data set (B). The dashed gray lines in both panels indicate *y = x*.

We now investigate another means of assessment that examines the relationship between structure accuracy and molecular flexibility. Figure 11 shows the mean RMSD between a given generated structure and the entire set of reference structures. This is directly compared with the mean RMSD between different reference structures for that compound. For the QM9 data set (panel A) we see mean reference-reference RMSDs ranging from about 1.0–1.9 Å. Mean generated-reference RMSDs are lower, indicating that our generated structures are high quality structures toward the center of the reference distribution. For the Drugs data set (panel B), the structures have higher mean reference-reference RMSDs, ranging from about 1.5–3.1 Å. The mean generated-reference RMSDs are again mostly lower than the reference-reference RMSDs, although we see a strong correlation between the two variables. This is unsurprising: for the more flexible targets, a broader range of structures will be possible, increasing both reference-reference RMSD and generated-reference RMSD.

**Figure 12:**
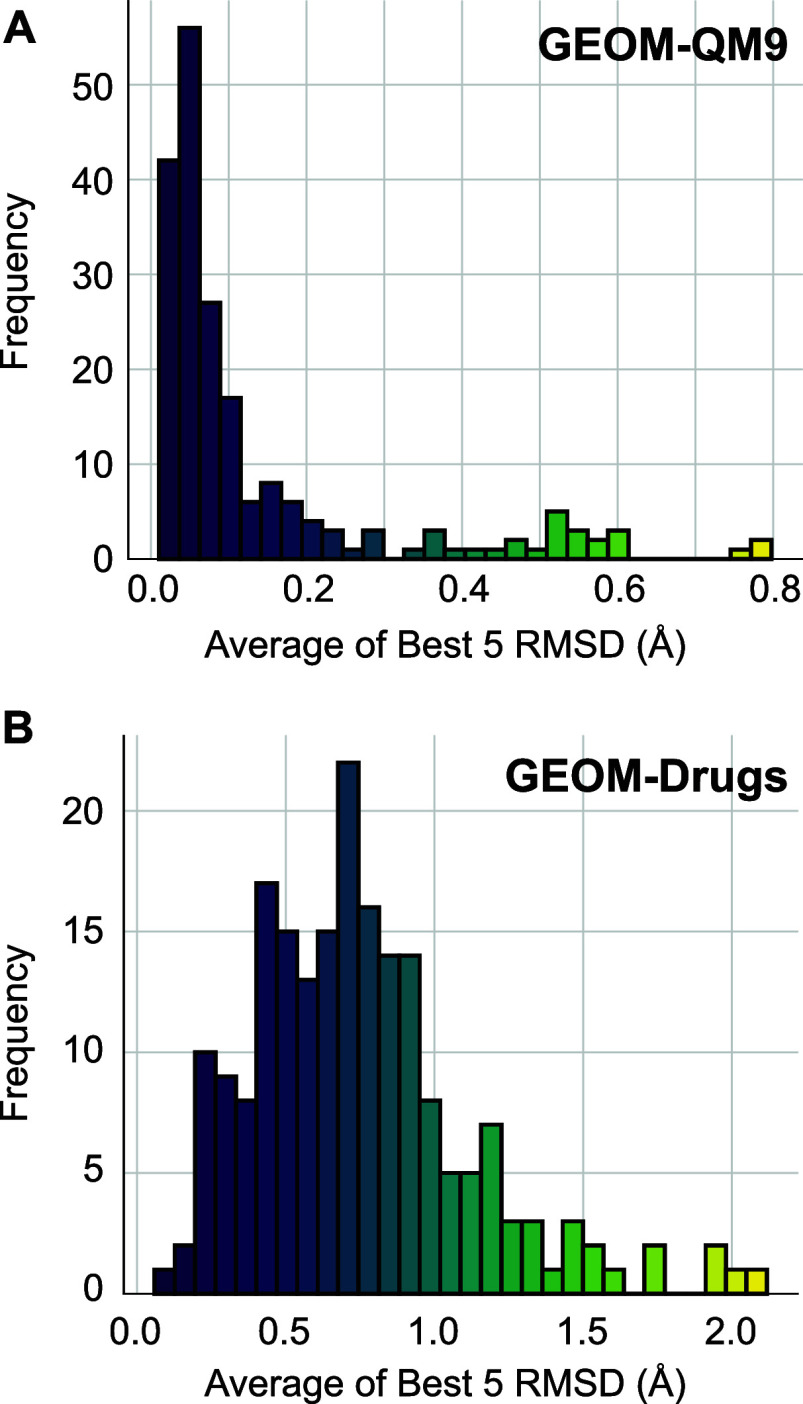
Comparison of the Best-5 RMSD Distribution between QM9 and Drugs Data sets. The top plot represents the QM9 data set, while the bottom plot represents the Drugs data set.

To evaluate how the best-case performance of the model is impacted by molecular properties, we built 200 structures for each molecule in the QM9 and Drugs test sets, and found the 5 best molecules with the smallest RMSDs to one of the target structures. Distributions of the Best 5 RMSD for QM9 and Drugs are shown in Figure 12. For QM9, this is sharply peaked at approximately 0.05 Å, indicating that near perfect matches are found for the vast majority of molecules in the data set. There are a small group of outlying molecules with higher RMSD values, extending to 0.8 Å. For Drugs, the distribution is peaked at roughly 0.7 Å, with the high end of RMSD reaching to 2.1 Å. Table 3 shows the correlation coefficients between molecular properties and the average of the best 5 RMSD values for each molecule. The correlation matrix for the Drugs data set reveals significant relationships between specific molecular properties and the average of the best 5 RMSD values, highlighting the challenges in accurately predicting conformations for drug-like molecules. A high positive correlation of 0.72 between the number of atoms and RMSD suggests that larger molecules with more atoms tend to have higher RMSD values, indicating increased difficulty in predicting their conformations. Similarly, the number of rotatable bonds shows a strong positive correlation of 0.55 with RMSD, emphasizing the challenge posed by molecular flexibility. The topological polar surface area (TPSA) also has a moderate positive correlation of 0.40, and also is extensive with the number of atoms. In that vein it is notable that the number of hydrogen bond donors does not show a strong correlation with RMSD. Finally, the logP shows a weak positive correlation with RMSD, indicating that more hydrophobic compounds are more challenging.

**Table 3:** Correlation Coefficients Between Molecular Properties and the Average of RMSD Values for All Test Dataset

Property	QM9 data set	Drug data set
Number of Atoms	0.22	0.72
Topological Polar Surface Area	0.26	0.40
logP	–0.06	0.29
Number of Hydrogen Donors	0.18	0.08
Number of Hydrogen Acceptors	0.17	0.38
Number of Rotatable Bonds	0.40	0.55

Similar analysis for the QM9 data set does not yield much insight, possibly due to the relatively low variation in best 5 RMSD values. The strongest correlation is seen with the number of rotatable bonds, which is intuitive, although this correlation (0.40) is limited by the smaller size of the molecules in QM9.

## 5 Limitations

While AGDIFF demonstrates promising results in molecular geometry prediction, several limitations and areas for future research warrant consideration:

(1) Environmental Conditions: The GEOM-QM9 data set represents molecular conformations optimized in vacuo at 0 K using density functional theory (DFT) calculations. Future studies should assess AGDIFF’s performance on data sets incorporating solvent effects and temperature-dependent conformational ensembles, such as those derived from ab initio molecular dynamics simulations. This extension would provide insights into the model’s capability to capture environmentally induced conformational changes and entropic effects, critical for understanding molecular behavior under physiological conditions.
(2) Method Dependence: The reference data in this study relies on specific computational methods (e.g., DFT for GEOM-QM9, CREST for GEOM-Drugs). Future research should evaluate AGDIFF’s performance across data sets generated using various levels of theory, including high-level ab initio methods (e.g., coupled-cluster, multireference methods) and classical force fields. Such comparisons would elucidate the model’s sensitivity to underlying energy surface representations and its transferability across different computational chemistry paradigms.
(3) Chemical Property Prediction: Our current evaluation metrics focus primarily on structural accuracy. Future studies should assess AGDIFF’s ability to predict chemically relevant properties such as electronic characteristics (e.g., dipole moments, polarizabilities), thermodynamic properties (e.g., conformational free energies), and spectroscopic observables (e.g., NMR chemical shifts, vibrational frequencies). This comprehensive evaluation would provide deeper insights into the model’s capacity to represent the underlying physics and chemistry of molecular systems.
(4) Rare Event Sampling: While the diffusion process in AGDIFF allows for conformational space exploration, it may face challenges in capturing rare events or high-energy transition states crucial in certain chemical processes. Previous work has shown that transition state structures can be generated using reactant and product states as input.^51^ Future work could focus on the prediction of transition state and other lower probability structures directly, without end point structures. This could potentially incorporate concepts from enhanced sampling techniques used in molecular dynamics, such as metadynamics^52^ or replica exchange,^53^ either during the inference process or in the generation of training data.

## 6 Conclusions

We introduced AGDIFF, a novel computational framework that leverages diffusion models for efficient and accurate molecular structure prediction. By extending the GeoDiff model with enhancements to the global, local, and edge encoders, AGDIFF demonstrates superior performance on both the GEOM-QM9 and GEOM-Drugs data sets compared to existing baselines. The incorporation of attention mechanisms, improved SchNet architecture, batch normalization, and feature expansion techniques enables AGDIFF to learn rich and expressive molecular representations while maintaining computational efficiency.

The experimental results highlight the effectiveness of AGDIFF in generating high-quality and diverse molecular conformations. On the GEOM-QM9 data set, AGDIFF achieves a mean COV-R of 93.08% and a mean MAT-R of 0.1965 Å, surpassing the GeoDiff baselines. Similarly, on the more challenging GEOM-Drugs data set, AGDIFF attains a median COV-R of 100.00% and a mean MAT-R of 0.8237 Å, demonstrating its ability to capture the conformational space of complex drug-like molecules.

The qualitative analysis of generated conformations, such as the Ramachandran plot for alanine dipeptide and the visualization of drug molecule predictions, further validates the quality and plausibility of the structures generated by AGDIFF. These results underscore the potential of AGDIFF to advance molecular modeling techniques and contribute to fields such as computational chemistry, drug discovery, and materials design.

Future work could explore the integration of AGDIFF with other molecular property prediction tasks, such as binding affinity, to provide a more comprehensive tool for drug discovery pipelines. Additionally, investigating the interpretability of the learned molecular representations and the incorporation of domain-specific knowledge could further enhance the model’s performance and applicability. Another potential direction is the incorporation of diffusion models as guiding forces in molecular dynamics simulations. The Flexible Topology method,^54^ developed by our laboratory, is one means of accomplishing this task, which would allow for external environments such as solvent or binding site atoms to influence the assembly of the diffusive particles as they evolve.

AGDIFF represents a step forward in molecular structure prediction, offering a powerful and efficient framework for generating accurate and diverse molecular conformations. By leveraging the strengths of diffusion models and introducing novel enhancements to the model architecture, AGDIFF has the potential to accelerate drug discovery efforts and advance our understanding of molecular systems.

## Data Availability

The software used to produce these results is available in the AGDIFF github repository (https://github.com/ADicksonLab/AGDIFF). The training data can be found at this link maintained by the Harvard dataverse (https://dataverse.harvard.edu/dataset.xhtml?persistentId=doi:10.7910/DVN/JNGTDF).

## Appendix A

### Training Configurations

AGDIFF employs a dual encoder network architecture and utilizes the diffusion model for both GEOM-QM9 and GEOM-Drugs datasets. The training configurations for both datasets are summarized in Tables 4 and 5.

**Table 4:** Training Configuration for GEOM-QM9

Parameter	Value
Hidden Dimension	128
Number of Convolutions	6
Number of Local Convolutions	4
Cutoff Distance	10.0
Activation Function	ReLU
Beta Schedule	Sigmoid
Beta Start	1 × 10^–7^
Beta End	2 × 10^–3^
Number of Diffusion Timesteps	5000
Edge Order	3
Edge Encoder	MLP
Smooth Convolution	False
Batch Size	64
Validation Frequency	5000 iterations
Maximum Iterations	1,000,000
Maximum Gradient Norm	10,000
Anneal Power	2.0
Optimizer	Adam
Learning Rate	1 × 10^–3^
Weight Decay	0
β_1_	0.95
β_2_	0.999
Scheduler Type	Plateau
Scheduler Factor	0.6
Scheduler Patience	10

**Table 5:** Training Configuration for GEOM-Drugs

Parameter	Value
Hidden Dimension	128
Number of Convolutions	6
Number of Local Convolutions	4
Cutoff Distance	10.0
Activation Function	ReLU
Beta Schedule	Sigmoid
Beta Start	1 × 10^–7^
Beta End	2 × 10^–3^
Number of Diffusion Timesteps	5000
Edge Order	3
Edge Encoder	MLP
Smooth Convolution	True
Batch Size	32
Validation Frequency	5000 iterations
Maximum Iterations	2,000,000
Maximum Gradient Norm	30,000
Anneal Power	2.0
Optimizer	Adam
Learning Rate	1 × 10^–3^
Weight Decay	0
β_1_	0.95
β_2_	0.999
Scheduler Type	Plateau
Scheduler Factor	0.6
Scheduler Patience	10

## Appendix B

### Equivariant Diffusion from Invariant Graph Neural Networks

Here we use the same technique implemented by Xu et al.^3^ to convert invariant graph neural network outputs to equivariant conformation gradients (ϵ_θ_). The outputs from the global and local encoders are sets of features for each node that are invariant to translation, rotation, and inversion, as they are determined solely using the distances between nodes. The global node features are denoted  for each atom *i*, and the local features are denoted . Separately for the global and local features, a scalar “edge strength” is determined for each edge. This is the output of a 3-layer multilayer perceptron (MLP) function that takes as input the concatenation of 1) the element-wise product of the node features for the source and target nodes corresponding to that particular edge, and 2) the edge features for that edge. The number of nodes input to each layer is [256, 128, 64], with a final output dimension of 1.

The global () and local ()

edge strengths are used to create equivariant conformation gradients (ϵ_θ_) as follows:

12

where *E*_*i*_ is the set of destination nodes for edges that originate in node *i*, *x*_*i*_ is the position of node *i* and *d*_*ij*_ is the distance between nodes *i* and *j*. A set of corresponding local transforms () are computed using the local edge strengths, . It is easy to show that these will be equivariant with changes in the atomic positions {*x*_*i*_}, as they only operate along inter-atomic displacement vectors, which rotate along with the reference frame.

## Footnotes

1 All results presented in Tables 1 and 2, except those for the AGDIFF and GADIFF models, are as reported in GeoDiff Xu et al.^3^ This includes results originally obtained from methods such as CVGAE,^46^ GraphDG,^47^ CGCF,^44^ ConfVAE,^48^ ConfGF,^49^ and GeoMol,^45^ consolidated and evaluated in the same experimental settings by Xu et al.^3^

2 The GADIFF^50^ method is another attention-enhanced SchNet encoder-based method that was reported between manuscript submission and publication and is included here for completeness.
